# Feasibility of procedures for a randomised pilot study of reduced exertion, high-intensity interval training (REHIT) with non-diabetic hyperglycaemia patients

**DOI:** 10.1186/s40814-020-00571-8

**Published:** 2020-02-19

**Authors:** Matthew Haines

**Affiliations:** Department of Allied Health Professions, Sport and Exercise, University of Huddersfield, Huddersfield, HD1 3DH UK

**Keywords:** Diabetes, Exercise, Feasibility, HbA_1c_, Interval training

## Abstract

**Background:**

Physical activity and exercise interventions to improve health frequently bring about intended effects under ideal circumstances but often fail to demonstrate benefits in real-world contexts. The aim of this study was to describe the feasibility of an exercise intervention (reduced-exertion, high-intensity interval training) in non-diabetic hyperglycaemia patients delivered in a National Health Service setting to assess whether it would be appropriate to progress to a future large-scale study.

**Methods:**

The intention was to recruit 40 participants from a single centre (specialist diabesity centre). Patients were eligible to take part if they were diagnostically defined as non-diabetic hyperglycaemic based on a glycated haemoglobin (HbA_1c_) value of 42–46 mmol mol. Study procedures including recruitment, occurrence of adverse events, intervention acceptability, and intervention adherence were used to assess feasibility.

**Results:**

Key criteria for progression to a larger study were not met. The study revealed several issues including patient eligibility, challenges to recruitment, patient consent, and poor clinician engagement. Furthermore, despite the simplicity and convenience of using HbA_1c_ to screen for diabetes risk, the process of accurately screening and case finding eligible patients was problematic. The small sample recruited for this trial (*n* = 6) also limits the interpretation of data, thus it is not possible to estimate the variability of intended outcomes to use in a formal sample size calculation for a full-scale trial. Some aspects of the intervention worked well. The acceptability of the exercise intervention and outcome measures met progression criteria thresholds and adherence was very high, with 97% of exercise sessions completed for participants that finished the study.

**Conclusions:**

Given the issues, the trial is not feasible in its current form. Yet, this preparatory stage of trial design pre-empted problems with the intervention that could be changed to optimise the design and conduct of future studies. Solutions to the issues identified in this study revolve around using a dedicated local recruiter with a strong relationship among the healthcare team and patients, using participant incentives to take part, and allowing for a longer recruitment period.

**Trial registration:**

ClinicalTrials.gov, NCT04011397. Registered 07 July 2019—retrospectively registered.

## Background

Encouraging individuals and populations to lead healthier lifestyles is inherently challenging because interactions between behavioural, biological, and environmental factors are complex. Therefore, attempts to tackle problems such as physical inactivity and obesity increasingly use complex interventions, conventionally defined as having several interacting components [1]. Consequently, it is important that sufficient initial development of interventions is undertaken to consider methodological bias and imprecision that could undermine eventual implementation in practice and to assess whether it is fundamentally appropriate and ethical to proceed with a large-scale trial [1, 2].

Medical Research Council (MRC) guidance highlights the requirement for greater attention to be given to the initial development of healthcare interventions because many interventions fail to demonstrate effectiveness in real-world contexts [3, 4]. This is relevant for exercise interventions because, although evidence for their efficacy in treating disease is strong, there is a dearth of evidence for their effectiveness in real-world settings [5]. Many studies intending to examine exercise effectiveness use laboratory-style methods and controls that would be impractical and uneconomical in real-world interventions [6]. These concerns are potentially decisive because effectiveness is what matters to patients and commissioners [5]. Thorough feasibility work is required as part of the preparatory stage of trial design to pre-empt problems with exercise interventions to ensure transferability into real-world practice [7]. The aims of such developmental work must compromise between scientific robustness and generalisability to routine real-world contexts [8].

The present feasibility work relates to a randomised pilot study to carry out a reduced-exertion and high-intensity interval training exercise programme within a specialist diabesity service in a National Health Service (NHS) Trust hospital. The expression ‘diabesity’ is used to describe the co-existence of the obesity and type 2 diabetes epidemics [9]. The service was initiated to address the growing prevalence of people with diabesity in the locality. The aim of the service was to manage the complex healthcare needs of people with diabesity and to simultaneously optimise glycaemic control and weight management [10]. An intermediary group of patients whose blood glucose levels are higher than normal but not high enough to be diagnosed as diabetic are referred to as having non-diabetic hyperglycaemia (NDH) or ‘pre-diabetes’, which are umbrella terms to describe intermediate hyperglycaemic states [11]. Hyperglycaemic blood glucose excursions in pre-diabetic states contribute to the development of macro- and micro-vascular disease risk [12, 13], and without intervention, it is estimated that up to 70% of patients in this group will eventually develop type 2 diabetes [14, 15]. Furthermore, more than 50% of those living in England, who are overweight (body mass index [BMI] > 25 kg m^2^) and ≥ 40 years old already have NDH [16]. Therefore, interventions—such as increased physical activity—for individuals who are diagnostically considered to have NDH are high priority, as they provide a substantial opportunity for preventing the future burden of diabetes on patients, the NHS, and the economy.

The evidence supporting exercise and physical activity as preventative or therapeutic treatments for obesity and diabetes is incontrovertible, and Tier 3 weight management services that include physical activity have been shown to be effective in patients with type 2 diabetes [17, 18]. However, a major challenge to the effectiveness of exercise is adherence [19], particularly in those with chronic disease [20] because adhering to the programme is a pre-requisite for the success of any intervention. The most commonly cited barrier to undertaking physical activity is perceived ‘lack of time’ [21, 22]. Consequently, there has been a recent interest in high-intensity interval training (HIT) which is characterised by brief periods of repeated very high-intensity exercise interspersed with longer periods of recovery. HIT is considered more time-efficient than traditional best evidence exercise guidelines which promote 150 to 300 min of moderate-intensity physical activity per week [23, 24]. However, critics of HIT have highlighted that the intensity of this type of activity may present an additional barrier for many, and that HIT is not appreciably more time-efficient compared to traditional exercise guidelines when inclusive of a warm-up, cool down, and recovery in-between high-intensity bouts [25, 26]. These concerns may be critical because the acceptability and tolerability of exercise interventions to those for whom they are intended is of primary importance [27]. More recently, attempts have been made to modify HIT to make it genuinely time-efficient [28, 29]. This approach is known as reduced-exertion, HIT (REHIT) and includes cycling for a total duration of 10 min, inclusive of 2 × 10–20-s cycle sprints against a braking force equivalent to 7.5% of body mass. Perceptual and affective (i.e. pleasure-displeasure) responses to such exercise are important, as these may predict future exercise behaviour [30, 31], and have been found to be no more negative compared to those associated with moderate-intensity continuous exercise [32].

At the time of developing, the proposal for this research, sparse academic literature had considered HIT or REHIT in a real-world context, or in patients with NDH. Two studies had used HIT in real-world settings, with group exercise sessions held outdoors in a public park [33] or group cycling sessions undertaken at a university gym [34]. Two further trials compared the effects of REHIT against an alternative exercise programme in patients with diabetes [29, 35]. Although participants were recruited from hospitals (in Norway and England, respectively), the interventions nevertheless took place in a laboratory environment. The HIT protocols and clinical endpoints used in these various studies differed, but two outcomes were of particular interest: cardiovascular fitness (maximal oxygen uptake) and blood glucose control. Although the effects of HIT on fitness were generally consistent, the effects on blood glucose control were less clear. Also, most were small studies making it difficult to draw inferences about the likely success of the planned trial in the specific NHS Trust used in this study with success likely to be sensitive to features of the local context, such as cultural diversity, the built environment, socioeconomic status, and public transport. Therefore, the primary aim of this study was to assess the feasibility and acceptability of implementing a REHIT intervention for NDH patients to inform decisions on whether to proceed to a full study. Specifically, the main objectives were to investigate the following: (1) feasibility of recruitment and retention, (2) intervention adherence, (3) acceptability of the intervention, (4) acceptability of randomisation, and (5) feasibility of collecting outcomes to assess clinical effectiveness.

## Methods

This study was a randomised pilot study.

### Participants

Patients with NDH, diagnostically defined as a glycated haemoglobin (HbA_1c_) value of 42–46 mmol mol (or 6.0–6.4%), were recruited from one UK hospital. This criterion aligns with recommendations from the World Health Organisation (WHO) [36].

### Inclusion

- Accessing the NHS Trust Weight Management Service (Specialist Diabesity Clinic)
- Aged between 18 and 65 years (inclusive)
- Diagnosed as NDH (using standard criteria)
- Male or female
- Any ethnicity
- Not currently partaking in a new structured exercise intervention
- Considered low or medium risk for exercise using standard risk stratification [37]

### Exclusion

- < 18 years and ≥ 66 years
- Currently partaking in a new structured exercise intervention
- Euglycaemic
- Diagnosed with type 1 or type 2 diabetes; taking insulin; history of end-stage liver or kidney disease, neuropathy or retinopathy; has hypertension that cannot be controlled by standard medication; has cardiovascular disease, or another contraindication to exercise
- Considered high risk for exercise using standard risk stratification [37]
- Unable to adequately understand verbal explanations and written information given in English (there were no funds available for translation services)

### Recruitment

The Chief Investigator formed a recruitment team at the hospital consisting several consultants, registrars, and a physiotherapist who all worked within the specialist diabesity service. A clinical trials assistant or clinical nurse specialist was not available to assist with recruitment for the trial. Patient eligibility to partake in the study was initially identified by the consultants via direct contact in the clinic and by registrars accessing patient records and contacting eligible patients by telephone. Eligible patients received a letter of invitation and a patient information sheet. If the patient was interested, they provided verbal consent for the Chief Investigator to contact them to arrange a mutually convenient time to meet at the clinic (hospital) to discuss the study. Eligibility to partake in the study was confirmed at this initial appointment and included further risk stratification using the American Heart Association/American College of Sports Medicine (ACSM) Health and Fitness Facility Pre-Participation Screening Questionnaire and the ACSM logic model for cardiovascular disease risk [37]. Patients considered high-risk were excluded from the study. The potential benefits, risks, and burdens associated with taking part in the study were explained, and participants were given the opportunity to ask questions before giving informed consent to take part. Recruitment was free from undue influence from the recruiters, and coercion or other inducements were not used. Funds were not available to reimburse participants for taking part or for travel expenses. Participants who consented had some baseline measures taken immediately by the Chief Investigator (blood pressure and body composition). At this point, arrangements were made for the participant to undergo an electrocardiogram (ECG) to screen for ischaemic heart disease, and a blood test to confirm HbA_1c_ prior to starting the trial. Dates were agreed for further baseline testing (a fitness test) and the start of the REHIT intervention, pending satisfactory results from the ECG and blood test. Eligible patients who were contacted and decided not to participate in the trial were asked to give a reason for declining which was recorded, if agreed by the patient. The recruitment period was fixed at 10 months.

### Sample size

As this study was aiming to assess feasibility, no formal sample size calculation was undertaken. However, a proposed sample size of 40 (20 per group) was considered adequate to meet the objectives of the study and was more than recommendations that suggest 12 patients per arm is appropriate [38, 39]. If the recruitment rate permitted, it was decided that the aim would be to increase the sample size (maximum 60) so that it would be consistent with the median sample size found in a review of 79 pilot studies [40]. Sample sizes within this range are recommended for a feasibility trial that aims to estimate variability (via standard deviation) which can then be used in a formal sample size calculation for a full-scale trial [41, 42]. Notwithstanding, there was no intention to power the feasibility study to test inferences about the intervention.

### Randomisation and concealment

The aim was to assign participants to one of two conditions using a random selection from a sequence of unmarked and opaque envelopes. The experimental group would receive usual care in addition to undertaking the REHIT intervention. The control group would simply receive usual care. Given the nature of the intervention, it would not be possible to conceal the treatment that participants were assigned to.

### Intervention (REHIT)

**Fig. 1:**
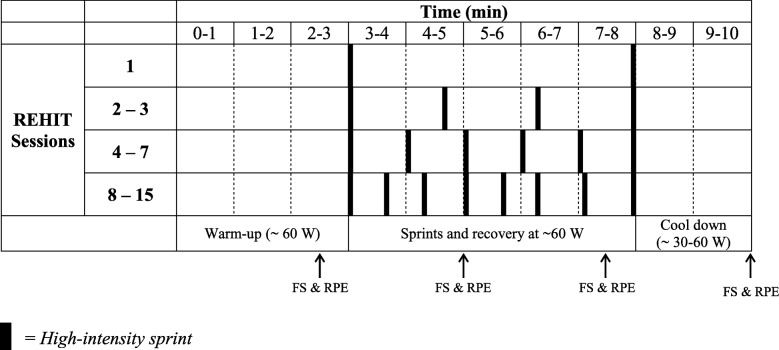
Schematic overview of the REHIT exercise intervention. Abbreviations: REHIT = reduced exertion, high-intensity interval training, FS = feeling scale; RPE = rating of perceived exertion

Participants were required to partake in a total of 15 sessions of REHIT. They were permitted to achieve this by completing two or three sessions per week until the target had been accomplished. Thus, the intervention lasted 5–7 weeks dependent on the frequency of sessions. This flexibility within the intervention was used to aid compliance and to more closely mimic how REHIT might be used in a real-life setting. All exercise sessions were performed on a magnetically braked cycle ergometer (Ergomedic 839E Digital, Monark, Vansbro, Sweden). Saddle height was adjusted for each participant to ensure close to full knee-joint extension (~ 170°) when the pedal was at the bottom of the cycle. Participants performed a total of 10 min cycling, inclusive of 2 (first session only), 4 (sessions 2 and 3), 6 (sessions 4 to 7), or 8 (sessions 8 to 15) × 5 s maximal effort ‘sprints’ against a braking force proportional to 7.5% of body mass. Exercise intensities in-between sprints were low (~ 60 W), and a warm-up (3 min at ~ 60 W) and cool down (2 min at ~ 30–60 W) were included within the 10-min session (Fig. 1). This protocol has been used previously and found to be tolerable [43].

Affective valence (i.e. pleasure-displeasure) and perceived exertion were assessed using the Feeling Scale (FS) [44] and Rating of Perceived Exertion (RPE) Borg scale [45], respectively. These constructs were measured to consider the perceptual response to and acceptability of REHIT, with FS and RPE recorded at 25%, 50%, 75%, and 100% of exercise completion (Fig. 1). Enjoyment was assessed using the Exercise Enjoyment Scale (EES) [46] 5 min post-exercise.

### Usual care

Members of a multi-disciplinary team including a consultant, diabesity specialist nurse, clinical psychologist, physiotherapist, and dietician worked together to promote patient self-management. Care was tailored to the specific circumstances of each patient after initial assessment and focussed on weight management, dietary education, behavioural therapy, and supported pharmacotherapy initiation as appropriate.

### Outcomes to assess feasibility

#### Progression criteria

A priori progression criteria were used to consider whether it would be appropriate to progress to a full-scale study. Based on other similar feasibility studies [47] these included (1) feasibility to recruit and retain participants, (2) intervention adherence, (3) intervention acceptability, (4) acceptability of randomisation processes, and (5) acceptability of planned outcomes to assess clinical effectiveness. Full details of criterion thresholds used to determine whether progression criteria had been met are included in Table 3.

#### Screening and eligibility

The screening rate was defined as the number of patients who had contact with the recruitment team and who were assessed for eligibility using inclusion and exclusion criteria. This included those who decided not to take part. Eligibility was determined by dividing the number of people screened by the number who met inclusion criteria.

#### Recruitment and reasons for not participating in the study

The recruitment team recorded why patients who met the inclusion criteria decided not to participate in the trial. The reason why patients who met the inclusion criteria and verbally consented to having their contact details given to the Chief Investigator and then subsequently decided not to participate were also recorded.

#### Adverse events

An adverse event was defined as any untoward occurrence that happened during the conduct of the study. All adverse events were recorded in the participant patient notes and were classified as serious or not, and attributable to the study or not, as per the ‘Decision Tree for Adverse Event reporting’ from the National Institute for Health Research, Clinical Research Network, Introduction to Good Clinical Practice Toolkit [48].

#### Retention rate

The retention rate was defined as the number of participants who remained in the study and did not drop out.

#### Completion rate

The completion rate was defined as the number of participants who completed the target number of 15 REHIT sessions.

#### Intervention adherence

Intervention adherence was calculated by summing the total number of participants and the target number of REHIT sessions (15 per participant) and comparing this against the actual completed sessions.

#### Intervention acceptability

Intervention acceptability was considered by measuring FS, RPE, and EES responses. These scales measure various theoretical constructs related to the perceptual response associated with exercise [44–46].

### Outcomes to assess clinical effectiveness

#### Cardiovascular fitness (peak oxygen uptake)

Cardiovascular fitness, or peak oxygen uptake (*V̇*O_2peak_), was measured at baseline and after the REHIT intervention. Participants cycled on a magnetically braked ergometer (Ergomedic 839E Digital, Monark, Vansbro, Sweden) starting at 20 W, with required power to continue cycling increased by 15 W every 1 min thereafter, until volitional exhaustion or until a pedal cadence of 50–60 rpm could not be maintained. Participants respired through a face mask connected via a sample line and volume transducer to an online gas analysis system (Metamax 3B, Cortex, Leipzig, Germany). Respiratory volume, expired oxygen and carbon dioxide, and respiratory exchange ratio were measured with *V̇*O_2peak_ taken as the highest value averaged over 10 s periods. Several additional criteria were considered to establish if *V̇*O_2peak_ had been reached: an observed plateau in the oxygen uptake curve, a respiratory exchange ratio of 1.15 or higher, and an RPE of 19-20 on the Borg scale [45].

#### Glycated haemoglobin

Blood samples were drawn at the beginning and end of the study by a trained phlebotomist using standard venipuncture after an overnight fast. Glycated haemoglobin (HbA_1c_) was measured by turbidimetric inhibition immunoassay (Roche, Basel, Switzerland) and was reported using the International Federation of Clinical Chemistry (IFCC) reference method [49].

#### Anthropometry and body composition

Stature and body mass were measured barefoot and wearing light clothes, using a stadiometer and Tanita® MC-180MA Multi-Frequency Body Composition Analyser (Tanita Europe, Netherlands), respectively. Body composition was estimated via whole-body bioelectrical impedance analysis (BIA) using a Bodystat® 1500 (COSMED Deutschland GmbH, Germany). With participants in a supine position, two source electrodes were placed on the dorsal surfaces of the foot and hand, with detector electrodes attached between the radius and ulna (styloid process) and between the medial and lateral malleoli. A painless, localised electrical current (~ 800 μA at a frequency of 50 kHz) was introduced with impedance to current flow between the source and detector electrodes determined based on the relationship between voltage and current (Ohm’s law). These relationships are used to quantify the volume of body water which is then converted to body density. Along with body mass, stature, gender, age, and ethnicity; body density was used to predict percentage body fat using the Siri equation [50]. BIA was selected as a non-invasive, safe, and easy means to predict body composition since other methods were not practical in the hospital clinic. BIA has been shown to underestimate body fat percentage by approximately 2% compared to Bod Pod® (air displacement plethysmography) but has been shown to have excellent reliability under standardised conditions [51]. Prediction of body fat percentage may be a more useful predictor of morbidity and mortality risk than the commonly used BMI [52, 53].

#### Blood pressure

Systolic and diastolic blood pressures were measured using an automated digital blood pressure monitor (A&D Medical, USA) with the pressure sensor placed over the brachial artery just above the antecubital fossa. Measurement was taken in a seated position after participants had rested for 5 min, with the arm relaxed level with the heart.

### Analyses

No probability statistical analyses were undertaken because the feasibility work did not aim to make inferences from the data. However, basic descriptive statistics including central tendency (mean) and variability (standard deviation) of scores were recorded to better understand the distribution of primary outcomes. Descriptive statistics were also used to summarise the screening, retention, completion, and adherence data.

### Ethical approval and research governance

NHS ethical approval for the study was provided by the National Research Ethics Service Committee Yorkshire and The Humber (REC reference 15/YH/0226; IRAS project ID 167716), in addition to NHS Research Management approval from the relevant NHS Trust Research Committee (R&D ID 15/997).

## Results

### Sample characteristics

Participants (*n* = 5) were South Asian (3/5) or White (2/5), with a mean age of 46.4 ± 6.1 years and were mostly overweight (60% of the sample; mean BMI 26.3 ± 3.9 kg m^2^) (Table 1).

**Table 1:** Sample characteristics and changes in outcomes to assess clinical effectiveness after the REHIT intervention

	Pre-intervention	Post-intervention
Age (years)	46.4 ± 6.1	–
Ethnicity:		
Asian British	*N* = 3 (60%)	–
White British	*N* = 2 (40%)	
Mass (kg)	71.2 ± 15.5	71 ± 14.8
BMI (kg m^2^)	26.3 ± 3.9	26.2 ± 3.7
*V̇*O_2peak_ (ml kg min^−1^)	30.6 ± 4.6	33.8 ± 4.3
HbA_1c_ (mmol mol^−1^)	44 ± 1.6	43.2 ± 0.8
Fat mass (%)	16.2 ± 5.8	16.2 ± 6.1
Systolic BP (mmHg)	118.4 ± 14.9	118 ± 13.3
Diastolic BP (mmHg)	75.6 ± 8.9	74.2 ± 8.3

Note: Data are presented as mean ± standard deviations

*Abbreviations*: *BMI* body mass index, *BP* blood pressure, *HbA*_*1c*_ glycated haemoglobin A_1c_, *REHIT* reduced exertion high-intensity interval training, *V̇O*_*2peak*_ peak oxygen uptake

### Screening (eligibility) (progression criterion 1)

**Fig. 2:**
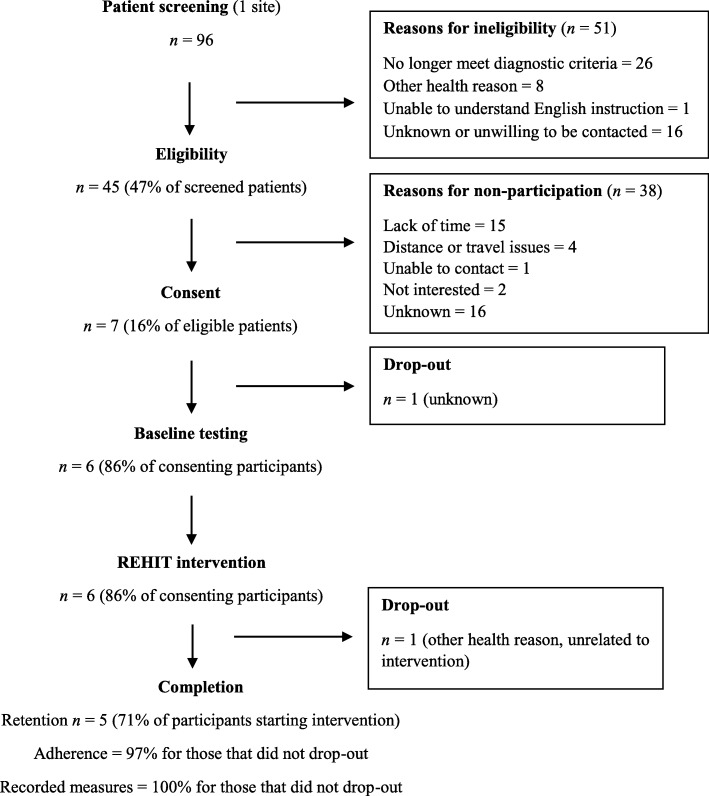
Participant flowchart. Abbreviations: REHIT = reduced-exertion, high-intensity interval training

A total of 96 participants were screened by accessing patient records or using direct contact in clinic. Figure 2 shows the flow of participants throughout the trial. Of the 96 patients identified, 51 (53%) were not eligible to partake in the trial, with two main reasons for this. First, 16 patients were unwilling to be contacted by the Chief Investigator or did not want to receive further information from the recruitment team after being provided with initial verbal information. Second, 26 participants (27% of all the participants screened) were no longer within the diagnostic range for NDH (i.e. 42–46 mmol mol) since their original diagnosis, with blood glucose control either having moved into the overt type 2 diabetes range or returned to euglycaemia (sometimes following lifestyle change). The remaining 45 patients received information about the trial.

### Recruitment and reasons for not participating in the study (progression criteria 1 and 4)

Due to insufficient recruitment, the inclusion of a control group was not feasible. As such, it was not possible to apply randomisation procedures and the control arm of the study was abandoned. The consent rate was 16% (7 out of 45 eligible patients), with ‘lack of time’ cited as the most common reason for not partaking (15 out of 38, 39% of eligible participants) (Fig. 2). Explanations for the poor recruitment were numerous. Many patients were reluctant to partake in the trial or were unwilling to be contacted, as mentioned above. Again, the number of eligible patients from those identified through screening was reduced due to patients no longer meeting inclusion criteria. Also, the recruitment team (single site) failed to initiate screening for eligibility at the predicted rate, especially during direct contact. Reasons for this included extremely busy clinics, insufficient frequency with which consultants see patients with chronic illness for lifestyle change advice in primary and secondary care, and the fact that ‘pre-diabetes’ may not be considered a serious illness by patients. A total of seven participants volunteered to take part in the study (7% of those screened, 16% of eligible patients), with one participant withdrawing before baseline testing (Fig. 2). Randomisation procedures were not feasible due to low recruitment, with the remaining six participants assigned to the REHIT intervention group.

### Adverse events (progression criterion 1)

No adverse events were reported during, or because of, the REHIT intervention.

### Retention rate (progression criterion 1)

The retention rate was 71% for patients that consented to partake in the study (2 out of 7 participants dropped out). However, one participant withdrew before baseline testing. The retention rate for participants who started the REHIT intervention was 86% (5 out of 6 participants completed).

### Completion rate (progression criterion 2)

The completion rate was based on the number of participants who completed the target number of 15 REHIT sessions. Of the 5 participants who completed the study, 3 participants managed 15 out of 15 sessions within 5–7 weeks, 1 completed 15 sessions > 7 weeks, and 1 participant completed 13 sessions only. Therefore, completion rate was 57% based on the seven participants that provided consent to partake in the study (4 out of 7), and 80% based only on the participants who did not formally drop-out of the study (4 out of 5).

### Intervention adherence (progression criterion 2)

Intervention adherence was calculated by summing the total number of participants and the target number of REHIT sessions and comparing this against the actual completed sessions. Therefore, the maximum number of sessions possible was 105 (7 participants who consented to partake × 15). As such, the completion rate was 72% (76 out of 105 sessions completed). However, the completion rate was much higher based on the five participants that did not drop-out of the trial (73 out of a possible 75 sessions completed, 97%).

### Intervention acceptability (progression criterion 3)

Acceptability of the intervention was good, with in-task pleasure and perceived exertion measured during REHIT using the FS and RPE scales, respectively. Enjoyment was also measured after cessation of exercise using the EES. Table 2 summarises perceptual responses throughout the intervention. Aggregate peak FS values ranged between 1.4 and 2.6 units, where 1 is ‘fairly good’ and 3 is ‘good’. Similarly, aggregate peak RPE values ranged between 12.7 and 13.9 units, corresponding to ‘somewhat hard’ and between ‘somewhat hard’ and ‘hard’. Enjoyment was reported as between 5.1 and 6 EES units (between “quite a bit” and “very much”). Taken together, these data suggest that the intensity of the REHIT protocol was tolerable, associated with modest perceived effort and was enjoyable. These theoretical concepts were used as a proxy for intervention acceptability. For the participant that dropped out of the study prior to baseline testing, it was not identified if the intervention and trial procedures were deemed unacceptable.

**Table 2:** Intervention acceptability. Peak perceptual responses to REHIT throughout the intervention (*n* = 5)

	Value	Notes
FS		
Sessions 1–3	2.6 ± 0.8	Affective valence (i.e. pleasure-displeasure) was assessed using the single-item, 11-point Feeling Scale [44]. The FS uses a bipolar scale and ranges from – 5 ‘very bad’ to + 5 ‘very good’, with anchors designated for 0 (neutral) and all odd integers in-between.
Sessions 4–7	1.7 ± 0.7	
Sessions 8–15	1.4 ± 0.7	
Average	1.9 ± 0.6	
RPE		
Sessions 1–3	12.7 ± 2.1	Exercise exertion was monitored using a rating of perceived exertion using the 15-point Borg scale [45]. The scale ranges from 6 ‘no exertion’ to 20 ‘maximal exertion’ with anchors designated for all the odd integers in-between.
Sessions 4–7	13.7 ± 0.9	
Sessions 8–15	13.9 ± 0.7	
Average	13.4 ± 0.6	
EES		
Sessions 1–3	6.0 ± 0.8	Enjoyment was assessed using the single-item, 7-point Exercise Enjoyment Scale [46]. Anchors are given at every integer, ranging from 1 ‘not at all’ to 7 ‘extremely’.
Sessions 4–7	5.2 ± 1.1	
Sessions 8–15	5.1 ± 0.3	
Average	5.4 ± 0.5	

Note: Data are presented as aggregate mean ± standard deviations. Values were recorded at 25%, 50%, 75%, and 100% of REHIT bout completion, with peak values only (at 75%) reported here

*Abbreviations*: *EES* exercise enjoyment scale, *FS* feeling scale, *REHIT* reduced-exertion, high-intensity interval training, *RPE* rating of perceived exertion

### Outcomes to assess clinical effectiveness (progression criterion 5)

Table 1 summarises sample characteristics and outcomes to assess clinical effectiveness, which would be designated as primary outcomes in the conduct of any larger-scale study. All participants that completed the intervention self-reported them to be acceptable. The feasibility study was not powered to test the effectiveness hypotheses associated with any planned main large-scale trial, and the sample size was very small. Nevertheless, there appeared to be a trend for increased cardiovascular fitness after the intervention. Mean *V̇*O_2peak_ increased from 30.6 ± 4.6 ml kg min^−1^ to 33.8 ± 4.3 ml kg min^−1^ (9% increase). There appeared to be no change in any of the other outcomes, including HbA_1c_.

### Summary of progression criteria

Three of the five criteria for progressing to a full-scale study were met, summarised in Table 3. Notably, recruitment and retention rates (progression criterion 1) were deemed to be ‘unlikely to be feasible’ with screening rates lower than anticipated. Although methods to increase recruitment were explored, it was felt that these issues could not be addressed sufficiently to justify progression without further feasibility work. The acceptability of the intervention and outcome measures (progression criteria 3 and 5, respectively) did meet the criterion threshold for ‘proceed’ and as such might be suitable in their current form for any future related studies.

**Table 3:** Summary of progression criteria for study procedures

Progression criteria	Assessment of whether criteria have been met	Outcome and decision
1. Feasibility to recruit and retain sufficient participants to meet targets within timeframe	Recruitment: percentage of eligible patients recruited; if > 30% recruited = proceed, if < 10% = unlikely to be feasible; if 10–30% = CI to consider feasibility of proceeding based on screening rate and possible steps to increase recruitment. Retention: percentage of participants retained; if > 80% = proceed, if < 60% = unlikely to be feasible, if 60–80% = CI to consider feasibility of proceeding based on available data and possible steps to increase retention.	Recruitment: 45 were eligible (96 were screened); 16% of eligible patients (7% of those screened) were recruited. CI decision was ‘unlikely to be feasible’ based on lower than anticipated screening rate. Retention: 71% of participants starting the intervention were retained. CI decision was ‘unlikely to be feasible’ based on lower than anticipated screening and recruitment rates.
2. Intervention adherence	Based on a hypothesised minimum dose; if > 80% = proceed, if < 70% = unlikely to be feasible, if 70–80% = CI to consider feasibility of proceeding based on available data.	Adherence was 72% (76 out of a possible 105 sessions completed) for all participants. Based on the 5 participants that did not drop-out, adherence was 97% (73 out of 75 sessions completed). CI decision was ‘proceed’ based on available data from participants that completed the study.
3. Intervention is acceptable to participants	Intervention acceptability was considered by measuring FS, RPE, and EES responses. Aggregate values for proceed were as follows: FS = > 0, RPE < 15; EES = > 3. Values below these thresholds = CI to consider feasibility of proceeding based on magnitude of values.	Acceptability of the intervention was good (see Table 2). Decision was ‘proceed’.
4. Randomisation processes acceptable to recruited participants	> 50% of recruited participants report agree about the acceptability of randomisation processes; the CI will apply discretion in judging whether this criterion has been met via discussion with participants.	Although planned, randomisation was not applied due to low recruitment. Control arm of the trial was abandoned.
5. Outcome measures acceptable to participants	Percentages of participants reporting acceptability of outcome measures on self-report questions. If > 80% = proceed, if < 50% = unlikely to be feasible, if 50–80% = CI to consider feasibility of proceeding based on available data and possible steps to increase acceptability.	Five out of 5 participants (100%) who completed the intervention recorded all measures and self-reported them to be acceptable. Decision was ‘proceed’.

Progression criteria based on Hawkins et al. [47]. *Abbreviations*: *CI* chief investigator, *EES* exercise enjoyment scale, *FS* feeling scale, *RPE* rating of perceived exertion

## Discussion

The findings revealed several issues relating to participant eligibility, recruitment, patient consent, and study duration that would need to be considered to optimise the design and conduct of future related studies. It was not possible, based on the data collected, to estimate variability for use in a formal sample size calculation for a future study and reinforces the need to answer questions relating to whether a study can be done in terms of estimating the rate of eligible people who are willing to participate [54]. Similar feasibility studies have highlighted that physical activity interventions can be challenging and resource-intensive with recruitment, retention, and participant dropout issues prevalent [47, 55–57]. As such, advancing to a large-scale study is not appropriate with the study in its current form because key progression criteria were not met. However, future research benefits from this developmental work, which underlines the role of feasibility studies undertaken to answer questions relating to whether a study can be done.

Six participants undertook baseline testing and commenced the exercise intervention. This occurred because of a range of challenges related to screening, eligibility, and consent, further compounded by a limited recruitment period (10 months) caused by unforeseen delays in acquiring ethical clearance for the study. Furthermore, 27% of potential participants identified via screening of patient records turned out to be ineligible due to no longer meeting the diagnostic criteria for NDH. That is, since the original diagnosis, HbA_1c_ values had increased into the diagnostic range for type 2 diabetes or decreased to normal levels. As such, screening for future diabetes risk using borderline HbA_1c_ values (42–46 mmol mol) represents a narrow window of opportunity, and patient recruitment needs to occur soon after diagnosis.

Part of the problem lies in the significant biological and assay variability associated with predicting blood glucose excursions. NDH encompasses several separate conditions, including impaired glucose tolerance (IGT) and impaired fasting glucose (IFG), which reflect different metabolic abnormalities. Evidence suggests differing progression rates to type 2 diabetes for these different measures of glycaemia [58–63]. The HbA_1c_ test may also result in both under-diagnosis (i.e. false negatives) and overdiagnosis (i.e. false positives) [11, 64]. Therefore, despite the simplicity and convenience of using HbA_1c_, the process of accurately screening and case finding eligible patients is inherently problematic.

Many participants were reluctant to participate with ‘lack of time’ cited as the most common reason (33% of those eligible). This is a known issue with physical activity lifestyle interventions [22]. Furthermore, the recruitment team failed to screen and identify eligible patients at the predicted rate. Reasons for this included busy clinics and insufficient patient contact time. A recent study using exercise in disease-free cancer survivors reported that recruitment staff did not approach people who ‘did not look like an exerciser’ [65]. Although speculative, it is possible that recruiters in the current study, consciously or otherwise, did not recruit patients who they thought were unlikely to agree to take part, or who they thought might find the intervention difficult or even unsafe. Hence, for any future study, it would be important to clearly define eligibility criteria, and to provide recruiters with relevant information and training about benefits and contraindications of physical activity [2].

A further general matter could be that patients and healthcare teams did not perceive NDH as a serious condition because it has been argued that current definitions of risk that include diagnosis of pre-diabetes create unsustainable burdens on healthcare systems and risk unnecessary medicalisation [11]. The rationale for treating NDH includes preventing or delaying the development and consequences of diabetes, and the consequences of pre-diabetes itself [66]. A recent meta-analysis identified that NDH is associated with an increased risk of coronary heart disease, stroke, and all-cause mortality in people with an HbA_1c_ value as low as 39 mmol mol [67]. Other research has demonstrated that lifestyle change including physical activity targets results in reduction in the incidence of diabetes [68, 69] and retinopathy [70].

Therefore, based on the findings of the present study, dedicated local recruiters should be considered essential for any future trials. This recommendation is based on several lines of reasoning. First, it would allow for improved screening, identification, and recruitment of participants to reach a sample size to sufficiently power the trial to meet the stated research objectives. Second, with appropriate training and support, dedicated recruiters could more accurately inform prospective participants about the benefits and risks of exercise, including the general risks associated with NDH, ensuring equity in access to healthcare. Third, having a dedicated recruiter would ameliorate the burden on busy clinicians.

Adherence to the exercise intervention was very high and no adverse events were reported. Also, the exercise intervention was acceptable to participants which is important because for REHIT to have any role in public health, it must be tolerable to those for whom it is intended. Hedonistic theories of motivation propose that above a certain intensity threshold a cascade of physiological responses negatively influence affective states [71], yet despite a gradual increase in exercise intensity across the intervention, such perturbations did not result in negative affective states (i.e. displeasure, or < 0 on the FS scale, Table 2). This could be key to optimise behaviour [72] and in-turn predict long-term exercise adherence [73, 74]. However, the timing of affective responses to REHIT is important because the intensity of exercise changes dramatically between periods of recovery and maximal effort sprints. In the present study measurements included periods shortly after completion of high-intensity sprints in an attempt to capture peak negative values but may have been different if they had been recorded *during* the high-intensity sprints. Nevertheless, when taken together, the adherence and affective responses suggest that REHIT might be a genuinely time-efficient, yet tolerable and sufficiently enjoyable approach to exercise for people with NDH.

Given the feasibility problems highlighted in this study, changes to improve any future study must be considered. Adjustments to the clinical context, including screening and recruitment strategies, within which the intervention is delivered, are required. As discussed, a dedicated local recruiter should be central to this. A significant number of eligible patients did not provide consent to take part in the study so it is likely that the perception of intervention acceptability is a concern. In hindsight, qualitative methods would have been a useful addition to further explore the acceptability of the intervention, as negative perceptions could potentially be assuaged by a knowledgeable recruiter. This may relate to perceived lack of time or other perceived burdens of the intervention such as travel and expense. Although the total time commitment (i.e. travel time, time in clinic, and exercise time) required by participants to attend exercise sessions was not recorded, it as likely to be substantial even though the exercise sessions were short. A greater understanding of the reasons for non-participation is needed because this information can be used to improve consent rate in future studies [2].

An alternative option is to use financial incentives to aid with recruitment and retention, as used in other exercise trials to improve health-related behaviours [75–78]. However, a financial incentive is a confound that may go beyond the confines of what is available in routine practice. Pragmatic studies of effectiveness in real-world contexts sacrifice internal validity for higher external validity and would be more appropriate [79]. Another option is to increase the complexity of the trial to include two components: intervention and incentive versus intervention alone [7]. A separate consideration is that many eligible patients might have found the location of the intervention (i.e. a hospital-based clinic) to be inconvenient or unacceptable. The rationale for running the intervention at the hospital was to co-locate clinical facilities with exercise prescription. By allowing clinical consultation and then commencement of exercise prescription to take place at the same place, it was hoped that patient care would be more seamless and that this might improve patient engagement. Conceivably, the intervention could be located at community or even home settings to improve the convenience for many patients.

The need to perform further studies examining the efficacy, acceptability, and longer-term adherence to REHIT as a practical ‘real-life’ intervention remains [29]. It is anticipated that changes to HbA_1c_ would be small because structured aerobic, resistance, or combined exercise training are associated with an HbA_1c_ decline of just − 0.67% in patients with type 2 diabetes [80]. Hence, any future study would need to be sufficiently powered to detect small, but clinically meaningful, changes in HbA_1c_. Again, it was not possible to determine if the processes for achieving randomisation would work smoothly. It is likely that some bias would be evident towards the REHIT intervention group, as patients might perceive this to be good for them. This could be lessened by including an active control group, rather than solely standard care.

Finally, the Chief Investigator for this study was a British Association of Sport and Exercise Sciences (BASES) Accredited Sport and Exercise Scientist and performed the exercise specialist role. It is not clear who could carry out this role within routine practice, although it has been proposed that a range of healthcare professionals could have an important role to play in treating physical inactivity related disease through exercise interventions [27]. This could include administering REHIT since the protocol is straightforward to communicate, requires minimal equipment, and could be incorporated into a multidisciplinary clinic, although specialist facilities for this purpose do not routinely exist. However, recent research has successfully used cycle ergometers developed for self-guided and public implementation of the REHIT protocol in workplace environments (university and hospital) [81]. Nevertheless, this would need to be considered alongside competing demands, and staff would need appropriate training and organisational support to do so [27, 82].

## Conclusions

Key criteria for progression to a full-scale evaluation were not met due to difficulties with recruitment, screening, and gaining the consent of eligible patients. The use of a dedicated local recruiter, participation incentives, and training of the healthcare team are solutions that must be balanced against considerations around the realities of building a pragmatic study into routine practice. Recruiting a sample sufficiently large to power the trial to meet the stated research objectives will also be important considering that clinical changes in HbA_1c_ are small, and that underpowered studies limit the ability to draw conclusions about the effect of interventions on health outcomes and are more likely to be abandoned. Therefore, it is recommended that the process of intervention development should continue. This is warranted because REHIT has the potential to be a genuinely time-efficient and acceptable exercise choice to counter some of the burdens of physical inactivity.

## Data Availability

The datasets used and/or analysed during the current study are available from the corresponding author on reasonable request.

## Abbreviations

ACSM: American College of Sports Medicine
BIA: Bioelectrical impedance analysis
BMI: Body mass index
ECG: Electrocardiogram
EES: Exercise enjoyment scale
FS: Feeling scale
HbA_1c_: Glycated haemoglobin
HIT: High-intensity interval training
IFG: Impaired fasting glucose
IGT: Impaired glucose tolerance
MRC: Medical Research Council
NDH: Non-diabetic hyperglycaemia
NHS: National Health Service
REHIT: Reduced exertion, high-intensity interval training
RPE: Rating of perceived exertion
*V̇*O_2peak_: Peak oxygen uptake (cardiovascular fitness)
